# AMPA Receptor Function in Hypothalamic Synapses

**DOI:** 10.3389/fnsyn.2022.833449

**Published:** 2022-01-31

**Authors:** Maria Royo, Beatriz Aznar Escolano, M. Pilar Madrigal, Sandra Jurado

**Affiliations:** Institute of Neuroscience CSIC-UMH, Alicante, Spain

**Keywords:** glutamatergic synapses, AMPAR-mediated synaptic transmission, AMPAR subunit switch, synaptic plasticity, hypothalamus, homeostasis, social behavior

## Abstract

AMPA receptors (AMPARs) are critical for mediating glutamatergic synaptic transmission and plasticity, thus playing a major role in the molecular machinery underlying cellular substrates of memory and learning. Their expression pattern, transport and regulatory mechanisms have been extensively studied in the hippocampus, but their functional properties in other brain regions remain poorly understood. Interestingly, electrophysiological and molecular evidence has confirmed a prominent role of AMPARs in the regulation of hypothalamic function. This review summarizes the existing evidence on AMPAR-mediated transmission in the hypothalamus, where they are believed to orchestrate the role of glutamatergic transmission in autonomous, neuroendocrine function, body homeostasis, and social behavior.

## Introduction

Glutamatergic α-amino-3-hydroxy-5-methyl-4-isoxazole propionic acid receptors (AMPARs) are central to regulate excitatory synaptic transmission in the central nervous system (CNS). Theirs characteristic fast kinetics differentiate them from the N-methyl-aspartate receptors (NMDARs), allowing a rapid depolarization of the postsynaptic membrane and making possible the high-fidelity propagation of electric signals between neuronal cells (Traynelis et al., 2010).

α-amino-3-hydroxy-5-methyl-4-isoxazole propionic acid receptors are concentrated at the postsynaptic membrane of excitatory synapses where are highly dynamic, moving in and out of synapses in both a constitutive and an activity-dependent manner. Changes in their number, subunit composition, post-translational modifications, and interaction with scaffolding and accessory proteins modulate the postsynaptic content of AMPARs, which allows a rapid tight control of the synaptic strength. These unique physiological properties make AMPARs a key regulatory element of synaptic plasticity, the ability of synapses to modify their responses according to the inputs they receive (recent reviews on this topic: Huganir and Nicoll, 2013; Herring and Nicoll, 2016; Nicoll, 2017; Diering and Huganir, 2018). The vast majority of groundbreaking studies on AMPAR structure, synthesis, trafficking, and function, have been performed in CA3-CA1 hippocampal synapses, a fundamental circuit for memory and learning, and an ideal model for structure-function studies (Neves et al., 2008). Furthermore, since long-term potentiation (LTP) was firstly reported by Bliss and Lomo (1973), multiple forms of synaptic plasticity have been described and most of them, despite its dependence on different receptor types or intracellular signaling cascades, rely on the ability of AMPARs to rapidly move in and out of synapses (Huganir and Nicoll, 2013; Herring and Nicoll, 2016; Nicoll, 2017; Diering and Huganir, 2018). However, most studies assaying AMPAR function have focused on a reduced number of brain areas, predominantly regions associated with high-order functions such as the hippocampus and the cortex, or midbrain areas involved in reward and goal directed behaviors (reviewed in Huang et al., 2009; Stuber et al., 2010; Lammel et al., 2014; Loweth et al., 2014; Bellone et al., 2021). In contrast, studies on the role of AMPAR-mediated transmission and plasticity in brain regions in control of autonomous, homeostatic and endocrine functions are scarcer. This lack in our knowledge prevents to attain a complete picture of the role of AMPAR function in the whole array of neuronal functions both high cognitive processes and, brain and body homeostasis maintenance.

The hypothalamus is the main brain structure involved in the regulation of hormone control due to its strong connection to the pituitary gland (Ulrich-Lai and Herman, 2009; Le Tissier et al., 2017). Embedded deep in the floor of the third ventricle, the hypothalamus constitutes an intricate structure comprised by distinct small nuclei of great cell heterogeneity. These features have hindered the unambiguous identification of hypothalamic synaptic and plasticity properties which are highly influenced by glutamatergic transmission (Iremonger et al., 2010), although many questions regarding glutamate receptors expression, composition and function remain to be elucidated in this brain area. This review summarizes the current knowledge on AMPAR function in the hypothalamus, and contextualizes it using the detailed mechanisms described in hippocampal synapses. Recent advancements on this topic expand our current view of the role of glutamatergic transmission and primarily AMPARs, as major drivers of metabolic processes, sexual and social behaviors, and emotional responses.

## AMPAR Structure and Function

### AMPAR Structure

α-amino-3-hydroxy-5-methyl-4-isoxazole propionic acid receptors are tetrameric ion channels formed by the assembly of homogenous or heterogeneous subunits constituted by GluA1, GluA2, GluA3, and GluA4 (Greger and Mayer, 2019). At the structural level, each subunit is composed by an extracellular domain, a transmembrane domain and an intracellular domain. In addition, two genetic processes: alternative splicing and RNA messenger edition, contribute to the diversity of AMPARs.

The extracellular domain of the receptor contains two important regions: The LIVBP (leucine/isoleucine/valine-binding protein-like domain) N-terminal domain (NTD) and the Ligand Binding domain (LBD). The NTD is the less understood motif, but it is believed to contribute to the receptor assembly and stability of specific receptor populations (Rossmann et al., 2011; Sukumaran et al., 2011; Herguedas et al., 2016). In addition, the NTD has been proposed to play a critical role in the contribution of AMPARs to synaptic transmission and long-term plasticity maintenance (Watson et al., 2017).

In contrast, the LBD has been extensively studied. This region is composed by two segments oriented toward the extracellular space and separated by the channel pore inserted in the plasma membrane. These two fragments form the specific pocket for glutamate sensing, which undergoes a fast conformational change upon ligand binding (Sakakura et al., 2019). In addition, this segment contains an alternatively spliced flip/flop exon and an R/G editing site, which defines the kinetics of receptor desensitization (Pei et al., 2009; Wen et al., 2017).

The transmembrane domain (TMD) is composed by four membrane segments (M) named M1, M2, M3, and M4, which constitute the channel pore and allow the entry of Na^+^ and to a lesser extent Ca^2+^ trough receptors comprised by particular subunits (Swanson et al., 1997; Cull-Candy et al., 2006; Isaac et al., 2007; Liu and Zukin, 2007). The most conserved region is formed by the four M3 helices which constitute the core structure of the channel pore. The M3 helices are connected by intracellular and extracellular loops linked to the LBD for activation gating (Taverna et al., 2000; Moore et al., 2013). In addition, the M4 segment is essential for subunit tetramerization, and trafficking (Herguedas et al., 2013; Salussolia et al., 2013; Gan et al., 2015, 2016; Greger et al., 2017). Furthermore, the M2 loop contains a Q/R editing site that controls specific processes like the retention of unedited subunits in the endoplasmic reticulum (ER) (Greger et al., 2003).

Finally, the carboxyl-terminal intracellular domain (CTD) located at the intracellular tail is the most variable domain between subunits and spliced variants. This region is involved in the regulation of receptor function including trafficking, synaptic anchoring and stabilization due to interactions with signaling complexes and postsynaptic scaffolds (Henley and Wilkinson, 2016; Díaz-Alonso and Nicoll, 2021). Most research efforts have aimed to elucidate the role of the CTD region in subunit-specific trafficking during basal and activity-dependent synaptic transmission (Huganir and Nicoll, 2013; Henley and Wilkinson, 2016; Díaz-Alonso and Nicoll, 2021). These studies have yielded controversial results, particularly in the case of the GluA1 CTD whose essential role during LTP (Hayashi et al., 2000), has been questioned by studies employing transgenic models and novel strategies for *in vivo* molecular manipulation (Kim et al., 2005; Granger et al., 2013; Díaz-Alonso et al., 2020).

α-amino-3-hydroxy-5-methyl-4-isoxazole propionic acid receptors are synthetized in the ER where inter-subunit interactions allow the ensemble of the receptors at the ER plasma membrane (Greger and Esteban, 2007; Greger et al., 2007; Schwenk and Fakler, 2020). AMPARs are built in a serial process that first involves the formation of dimers mainly determined by the NTD, and the subsequent association of dimers into tetramers driven by the arginine/glycine (R/G) editing site (Opazo and Choquet, 2011).

The editing of the R/G and the glutamine/arginine (Q/R) sites underlies the basis for the subunit composition of functional receptors. In particular, the switch of the arginine R743 to glycine in the LBD in any of the GluA1-GluA4 subunits influences the homo/hetero dimerization possibilities of the receptor (Greger et al., 2017). Interestingly, the edition of the glutamine Q586 to arginine at the M2 segment of the TMD occurs exclusively in GluA2 subunits, conferring the GluA2-containing receptors with an additional positive charge in the channel pore rendering it impermeable to Ca^2+^ (Seeburg, 1996; Seeburg and Hartner, 2003). This edition process impacts many aspects of the biosynthesis, assembly and transport of the receptors (Sommer et al., 1991; Greger et al., 2002, 2003; Wright and Vissel, 2012).

AMPAR physiology is further enriched by the diversity of the GluA1-GluA4 subunits which may exist in two different conformations determined by the incorporation of flip-flop variants, being the flip versions more permissive to ion entry upon glutamate binding (Seeburg and Hartner, 2003; Coleman et al., 2006; Pei et al., 2009). The combination of multiple checkpoints at the synthesis and post-transcriptional levels results on a majority of GluA1/GluA2 or GluA3/GluA2-containing AMPARs in the adult brain (Wenthold et al., 1996), whereas GluA4-containing receptors, primarily GluA2/GluA4 heterodimers, are prominent during embryonic development to be drastically reduced at early postnatal stages (Zhu et al., 2000). In addition, Q/R unedited GluA2 subunits are steadily displaced by Q/R edited GluA2 subunits to comprise 99% of the GluA2-containing receptors in the mature brain (Monyer et al., 1991; Pachernegg et al., 2015).

The exit of the receptor from the ER is a complex process with multiple quality control steps, which involve the refinement of the domains involved in glutamate binding, and the association to signaling and scaffold proteins (Greger and Esteban, 2007; Greger et al., 2007; Esteban, 2008; Parkinson and Hanley, 2018). Then, the receptors travel to the Golgi apparatus where AMPARs undergo post-translational modifications influencing their stabilization at the postsynaptic density, the modulation of their function and kinetics and the activation of intracellular signaling cascades that determines neuronal communication and input integration (Greger et al., 2017). The receptors travel through the trans-Golgi network and later enter into the endosomal recycling system ready to be inserted into the postsynaptic density (Hanley, 2010; Opazo and Choquet, 2011). Once they reach the plasma membrane and during the whole receptor life cycle, a variety of post-translational modifications (Mao et al., 2011) will modulate their function by fast signaling changes like phosphorylation, stability at the plasma membrane by palmitoylation, recycling and maintenance by sumoylation or protein degradation in an ubiquitin-dependent manner (reviewed in Lu and Roche, 2012).

### AMPAR Trafficking and Plasticity

Neural plasticity was observed for the first time by Terje Lomo and Tim Bliss in 1973 (Bliss and Lomo, 1973) in the hippocampus of anaesthetized rabbits, when they described how the delivery of electrical activity at high frequency led to a robust and long-lasting increase of the postsynaptic responses of those cells receiving the stimulus, in a phenomenon known as long-term potentiation (LTP). Almost two decades of the discovery of LTP in the hippocampus, it was exposed that the reversal process known as long-term depression (LTD) (Ito, 1989) was also possible at the model CA3-CA1 synapse (Dudek and Bear, 1992a). The phenomenon of long-term plasticity, particularly LTP, was soon regarded as a plausible cellular mechanism for learning and memory, thus concentrating great efforts to unveil the molecular underpinnings involved in the induction and stabilization of long-term changes of synaptic strength. Cumulative evidence over several decades of intensive research has led to a prominent working hypothesis which postulates AMPARs as the main player of the molecular changes occurring during synaptic plasticity, largely due to their dynamic subunit composition and trafficking properties (Huganir and Nicoll, 2013; Herring and Nicoll, 2016; Nicoll, 2017; Diering and Huganir, 2018).

After the exit from the Golgi apparatus, AMPARs may be driven to the plasma membrane at extra-synaptic sites to later diffuse to the synaptic membrane or to be accumulated in recycling compartments where can be further recycled or incorporated at synaptic or peri-synaptic locations (Park et al., 2004; Penn et al., 2017). Both trafficking pathways seem to be regulated in an activity-dependent and subunit-specific manner which highly influences the properties of synaptic transmission and plasticity. In this regard, AMPARs containing short C-tails like GluA2 and GluA3 seem more likely to traffic in and out of the membrane in a constitutive manner, during a process that ensures the maintenance of AMPARs at the postsynaptic membrane (Shi et al., 2001). On the other hand, subunits exhibiting long C-tails as in the case of GluA1 and GluA4 subunits, are accumulated at early and recycling endosomes where they are available to be rapidly recruited in an activity-dependent manner (Shi et al., 2001). This complex scenario results in the fine regulation of the receptor transport in which specific subunits can be incorporated at distinct membrane locations at critical time points (Diering and Huganir, 2018).

α-amino-3-hydroxy-5-methyl-4-isoxazole propionic acid receptor C-tails also modulate the binding to regulatory subunits like TARPs or cornichons, transmembrane proteins that interact with AMPARs, modulating channel conductance and facilitating receptor biosynthesis and transport (Tomita et al., 2003; Greger et al., 2017; Kamalova and Nakagawa, 2021). These regulatory proteins are selectively targeted to plasma membrane microdomains enriched with specific phosphoinositides (PIP), which highly influence AMPAR localization. In this sense, the balance of PIP3/PIP2 levels at the plasma membrane plays an important role in receptor stability during plasticity. As such, the increment of PIP_3_ levels favors AMPAR insertion and facilitates LTP maintenance (Arendt et al., 2010) whereas reduced PIP_3_ levels lead to receptor endocytosis (Jurado et al., 2010b). In fact, PI3K and PTEN, the two main regulators of PIP_3_/PIP_2_ levels have been identified to play a critical function in plasticity events (Man et al., 2003; Jurado et al., 2010b).

Although AMPARs are primarily synthetized at the somatic ER, the required machinery for receptors biosynthesis has also been found in dendrites, where local transduction and translation rapidly occur in response to neuronal activity (Krug et al., 1984; Steward and Schuman, 2001; Ju et al., 2004). During both somatic and dendritic synthesis, receptors are transported through the cellular cytoskeleton in a process that requires the participation of motor proteins, either for constitutive recycling or activity-driven transport. AMPARs are capable of interacting with the microtubule-enriched cytoskeleton, predominantly present in dendrites, through the C-tail PDZ domain which facilitates the interaction with GRIP1, thought to act as a prominent link to motor proteins (Setou et al., 2002). Other proteins involved in the microtubular transportation of AMPARs are KIF1 (Shin et al., 2003), liprin-α (Hales et al., 2001) or GIT1 (Ko et al., 2003). Within dendritic spines, microtubules are replaced by an actin-enriched cytoskeleton (Hanley, 2014), which redirects the transport of the receptors to the plasma membrane. This step involves a specific ensemble of scaffolding proteins involving 4.1N, RIL or SAP9, motor proteins like Myo Vb (Wang et al., 2008), Myo Va (Correia et al., 2008) and MyoVI (Osterweil et al., 2005), and small GTPases from the Rab family (Rab11, Rab8) (Brown et al., 2007) and its accessory FIP proteins (Wang et al., 2008; Royo et al., 2019). Activity-dependent insertion of receptors at the plasma membrane is achieved by an exocytic process orchestrated by the interaction of specific SNARE proteins (Jurado, 2014; Madrigal et al., 2019). Synaptotagmin-1 and -7, complexin-2, syntaxin-3, or SNAP-47 have been shown to participate in the incorporation of AMPARs to the plasma membrane in response to NMDAR activation (Ahmad et al., 2012; Jurado et al., 2013; Wu et al., 2017). Once at the plasma membrane, AMPARs can laterally diffuse until being stabilized at postsynaptic regions through the interaction with scaffolding proteins, primarily from the MAGUK family (Membrane-Associated Guanylate Kinase) like PSD95 or PSD93, via indirect interactions with auxiliary proteins such as TARPs (Díaz-Alonso and Nicoll, 2021). Additionally, proteins involved in the insertion of receptors like GRIP1 and NSF also participate in AMPAR membrane stabilization (Braithwaite et al., 2002; Greger et al., 2017; Bissen et al., 2019).

AMPAR removal from the postsynaptic membrane is mediated by a classic clathrin-mediated endocytic process that may occur in a constitutive or in an activity-dependent manner, as it has been described during LTD induction (Malenka and Bear, 2004). Although GluA2 subunits have been proposed to be major drivers of receptor internalization, their exact role is not yet fully understood. These subunits may facilitate the interaction with drivers for protein endocytosis such as the clathrin adaptor AP2 (Fiuza et al., 2017). A plausible hypothesis proposes a mechanism in which AP2 competes with NSF for receptor binding (Lee et al., 2002), driving endocytosis as a result of an increase in the fraction of AP2 bound to the GluA2-PDZ domain. In addition, AKAP150, PSD95, PKA, PICK1, and small GTPases like Rab5 or Arf1 are also required in the constitutive and activity dependent internalization of AMPARs (Beattie et al., 2000; Brown et al., 2005; Bhattacharyya et al., 2009; Han et al., 2009; Citri et al., 2010; Jurado et al., 2010a; Hanley, 2018; Hausser and Schlett, 2019; Cheng et al., 2020).

After endocytosis, receptors traffic to early endosomes, also known as sorting endosomes, and targeted to different endosomal pathways to either enter the recycling system or be degraded by lysosomal or proteasomal pathways (Parkinson and Hanley, 2018). AMPARs sorting either involves entering recycling endosomes to be inserted into the plasma membrane, or be retrograde transported to the trans-Golgi network for post-translational modification (Parkinson and Hanley, 2018). On the other hand, receptors may be targeted for degradation by ubiquitination (Widagdo et al., 2017), an enzymatic reaction achieved by the coordinated and sequential action of the E1, E2, and E3 proteins, which target specific receptors to lysosomes. The proper balance of recycling and degradation pathways is critical for regulating AMPAR number and determines synaptic transmission and plasticity, particularly LTD maintenance (Fernández-Monreal et al., 2012; Widagdo et al., 2015).

## Glutamatergic Transmission in the Hypothalamus

The hypothalamus acts as a central integrator of neuronal and endocrine information controlling hormone secretion, homeostatic functions, and shaping complex behaviors such as social interactions (Saper and Lowell, 2014). The hypothalamic system can be divided in three main regions: periventricular, medial, and lateral in a coronal plane. These regions are composed by small and dispersed neuronal clusters that comprise distinct morphological and functional nuclei such as the paraventricular nucleus (PVN), supraoptic nucleus (SON), suprachiasmatic nucleus (SCN), dorsomedial hypothalamus (DMH), ventromedial hypothalamus (VMH), lateral hypothalamus (LH), the arcuate nucleus (ARC) and the retrochiasmatic area (RHC). These nuclei are highly interconnected providing a communication hub between the CNS, the autonomic nervous system and the endocrine system.

Arguably, the PVN and the SON are among the most studied hypothalamic nuclei mainly due to their prominent involvement in the hypothalamic-neurohypophysial axis as major sources of oxytocin (OXT) and arginine-vasopressin (AVP) (Swanson and Sawchenko, 1983; Brown, 2016; Qin et al., 2018). Neurons in hypothalamic regions are classically categorized in magnocellular or parvocellular, with specific functional and morphological features (Swanson and Sawchenko, 1983; Luther and Tasker, 2000; Luther et al., 2002; Tasker et al., 2020). The magnocellular system is formed by large neurons that produce OXT or AVP mainly released to the peripheral nervous system at the level of the pituitary gland. Conversely, the parvocellular system is composed by smaller neurons primarily connected to the CNS, the brainstem and the spinal cord (Swanson and Sawchenko, 1983). However, new advancements on anatomical and genetic techniques have enabled to revisit the connectivity and functional properties of magno and parvocellular neurons (Althammer and Grinevich, 2017). A recent study revealed that oxytocinergic magnocellular neurons can innervate forebrain areas like the central amygdala (Knobloch et al., 2012), and SON oxytocinergic parvocellular cells were observed to directly innervate magnocellular neurons (Eliava et al., 2016). Furthermore, two studies by Romanov et al. (2017) and Xiao et al. (2017) reported distinct types of oxytocinergic neurons, according to their expression of genetic markers and ability to modulate dopaminergic function, suggesting that the classical cellular classification in magno and parvocellular neurons needs to be reconsidered.

Although, the hypothalamus is mainly recognized as a neuropeptidergic hub, communication among hypothalamic nuclei is greatly facilitated by glutamatergic-mediated transmission (Van Den Pol et al., 1990; Meeker et al., 1993, 1994a; Van den Pol and Trombley, 1993; Brann, 1995; Hrabovszky and Liposits, 2008; Iremonger et al., 2010). However, the properties of excitatory transmission and plasticity in this brain area has been scarcely studied in contrast to the hippocampus.

### AMPARs and NMDARs Expression in Hypothalamic Nuclei

Classical *in situ* hybridization studies in the rat brain revealed widespread expression of AMPA, kainate, and NMDA receptor mRNA in the hypothalamus, at similar levels than in the cortex and the hippocampus (Van Den Pol et al., 1994; Meeker et al., 1994a; Herman et al., 2000; Eyigor et al., 2001; Ziegler et al., 2005). The role of glutamate-mediated transmission all along the hypothalamus has been further supported by receptor autoradiography, electrophysiology, and calcium imaging experiments which demonstrated distinct intracellular calcium dynamics in response to different glutamate receptor agonists (Stern et al., 1999).

A detailed map of glutamate receptors expression across the hypothalamus has been carried out in two different animal models: rat and guinea pig. In the rat hypothalamus, Eyigor and colleagues investigated the expression of ionotropic glutamate receptors by *in situ* hybridization detecting high levels of GluA1, GluA2, GluK2, GluN1, GluN2A, and GluN2B across the different hypothalamic nuclei (Eyigor et al., 2001). GluA1 and GluA2 subunits predominance in the rat hypothalamus differentiated from the observations of Waremburg and colleagues in the guinea pig hypothalamus. In this case, the predominant subunits were GluA2 and GluA3 and to a lesser extent GluA1, whereas GluA4 immunoreactivity was very low in all the researched regions (Warembourg and Leroy, 2002). Furthermore, the mandatory NMDAR subunit, GluNA1 was detected throughout the rat hypothalamus (Van Den Pol et al., 1994; Ziegler et al., 2005). Pioneer histological studies also reported low to moderate expression of group I metabotropic glutamate receptors (mGluR1 and mGluR5) in hypothalamic neurons (van den Pol, 1994; Van Den Pol et al., 1994; Kocsis et al., 1998).

**FIGURE 1:**
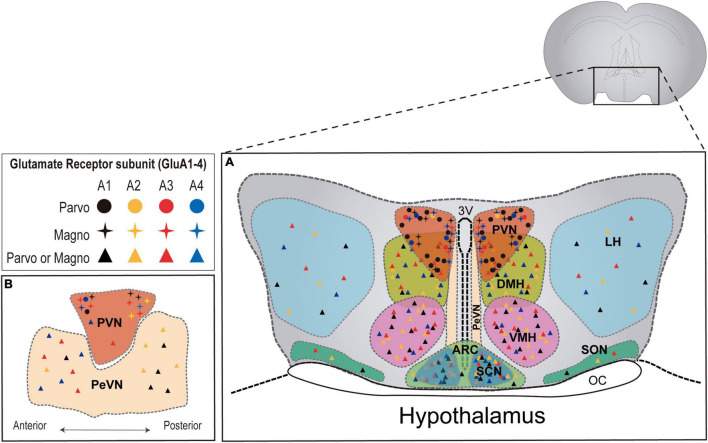
AMPAR subunit distribution across distinct hypothalamic nuclei in the rat brain. **(A)** Coronal plane of the hypothalamus showing the different hypothalamic regions. PVN = paraventricular nucleus; DMH = dorsomedial hypothalamus; VMH = ventromedial hypothalamus; LH = lateral hypothalamus; SON = supraoptic nucleus; PeVN = periventricular nucleus; SCN = suprachiasmatic nucleus; ARC = arcuate nucleus. **(B)** Sagittal plane of hypothalamic nuclei. AMPAR subunit abundance is represented for each nucleus according to available literature in the rat brain (Van Den Pol et al., 1994; Eyigor et al., 2001). Detailed information regarding AMPAR subunit abundance in magno and parvocellular neurons is only available for the PVN (Herman et al., 2000).

Interestingly, AMPAR subunits seem to exhibit region- and cell- specific expression patterns (Figure 1). As such, GluA1/2/4 are abundant at preoptic areas, whereas at the tuberal level (e.g., ventromedial and dorsomedial nuclei) the higher expression corresponds to GluA1/2/3 subunits. Furthermore, GluA1 and GluA2-containing receptors are predominant in the mammillary nuclei, where GluA3 and GluA4 subunits appear at lower levels (Van Den Pol et al., 1994). Taking into consideration that subunit composition is critical for AMPAR functionality, influencing multiple aspects of their biology from biosynthesis, transport, kinetics, to protein interactions, region- and cell-specific expression of glutamate receptors suggests multiple modes of glutamatergic transmission in the hypothalamus, which may underlie and modulate its various central and neuroendocrine functions (Brann, 1995).

### AMPAR- and NMDAR- Mediated Transmission in Hypothalamic Neurons

As aforementioned, hypothalamic function is highly influenced by glutamatergic transmission (Van Den Pol et al., 1990; Boudaba et al., 1997; Marty et al., 2011). As such, OXT- and AVP -expressing cells, located in the SON and PVN nuclei, receive dense glutamatergic innervation (Van Den Pol et al., 1990; Meeker et al., 1993) and express both postsynaptic AMPARs and NMDARs (Gribkoff and Dudek, 1990; Gribkoff, 1991; Wuarin and Dudek, 1993; Yang et al., 1995) which are believed to influence their firing patterns and ability to release OXT and AVP. Pulsatile hormone release involves secretion events which follow regular temporal patterns achieved by bursting synchronization (Poulain and Wakerley, 1982; Belin and Moos, 1986). Bursting synchronization is differentially regulated in hypothalamic magnocellular and parvocellular neuroendocrine cells (Eliava et al., 2016; Xiao et al., 2017; Lewis et al., 2020) consistent with their distinct expression of voltage gated ionic channels, permeable to either Ca^2+^ or K^+^ (Luther and Tasker, 2000). As such, AVP neurons have been shown to transition from slow and irregular patterns of activity to a phasic bursting, consistent with burst and inter-burst intervals of 20-30 sec, whereas OXT neurons commonly transition from irregular to continuous firing patterns (Dyball et al., 1991; Tasker and Dudek, 1991). The generation of different activity patterns, although highly dependent on intrinsic excitability properties, is also determined by glutamatergic synaptic inputs (Armstrong et al., 2010). A prominent example are the SON magnocellular neurons, which receive multiple excitatory inputs from the organum vasculosum lateral terminalis, olfactory nuclei, and the dorsal hypothalamus integrated by the activation of GluA1-4-containing AMPARs (Petralia and Wenthold, 1992; Ginsberg et al., 1995). Indeed, these neurons show linear current-voltage relations, and are capable of eliciting fast action potentials (Tasker and Dudek, 1991) which rapidly adapt to meet the requirements of hormone release.

Moreover, hormonal secretion at neurohypophysial terminals is largely regulated by glutamatergic activity onto both, OXT and AVP magnocellular neurons. *In vivo* studies have shown that bursting activity of magnocellular neurons promotes OXT release required for lactation in a NMDAR and AMPAR-dependent manner (Hu and Bourque, 1992; Lambert et al., 1993; Parker and Crowley, 1993; Moos et al., 1997). Similar to OXT cells, AVP neurons unsynchronized phasic activity (Poulain and Wakerley, 1982) requires NMDARs activation (Nissen et al., 1994; Moos et al., 1997). Interestingly, electrophysiological studies in rodent models (Stern et al., 1999; Luther and Tasker, 2000; Eliava et al., 2016; Xiao et al., 2017; Lewis et al., 2020) identified that OXT neurons in the SON displayed larger AMPAR-mediated miniature EPSCs (mEPSCs) and faster decay kinetics than AVP neurons (Stern et al., 1999). In both cell types, AMPAR-mediated synaptic responses showed inward rectification, although this feature was more pronounced in OXT neurons, which also displayed larger calcium permeability, likely due to a low expression of GluA2-containing receptors in these cells (Stern et al., 1999).

Even though early *in vivo* work suggested NMDAR contribution to synaptic responses was larger in AVP neurons and practically inexistent in OXT neurons (Nissen et al., 1994, 1995; Yang et al., 1994; Richardson and Wakerley, 1997), later *ex vivo* studies identified clear NMDAR-mediated currents in OXT neurons (Stern et al., 1999). Interestingly, NMDARs have been shown to inhibit OXT release in the posterior pituitary while, a combination of AMPARs and mGluRs activation promotes somatodendritic OXT release (Pampillo et al., 2001). Although the molecular details underlying these differences remain to be elucidated, cell-specific glutamatergic modulation of hypothalamic neurons may provide hypothalamic circuits with the ability to display various firing patterns in response to similar physiological stimuli, likely through a mechanism influenced by differences in AMPAR and NMDAR subunit composition in both OXT and AVP neurons, as it has been shown in principal and inhibitory neurons in other brain regions (Geiger et al., 1995).

### Hypothalamic Plasticity

The functional and molecular properties of glutamatergic plasticity in the hypothalamus have been understudied (Le Tissier et al., 2017) in comparison to other brain areas, such as the hippocampus. The heterogeneous composition of the hypothalamus, comprised by various cell types embedded in intricate nuclei lacking a laminar organization, posed major technical challenges that are now beginning to be overcome by novel circuit and functional mapping strategies as well as cell-specific genetic manipulations. Furthermore, the synaptic properties of hypothalamic neurons seem to differ from cells in other areas preventing a straightforward implementation of traditional plasticity protocols. As such, glutamate transmission onto PVN neurons exhibit short-term depression in response to high frequency stimulation (greater than 2 Hz), suggesting that glutamatergic transmission in the hypothalamus may shows higher fidelity at lower rates of synaptic activity (Marty et al., 2011). This effect was described as a mostly presynaptic phenomenon involving glutamate vesicle depletion, which reduced transmission efficacy upon high frequency stimulation (Marty et al., 2011). Although not completely definitive, these findings strongly suggest that high frequency protocols, classically used to elicit long-term potentiation in the hippocampus, may result in synaptic depression in the hypothalamus.

Similarly, a form of presynaptic short-term potentiation has been observed in Agouti related protein (AgRP)-expressing cells and propio-melanocortin (POMC) neurons, from the arcuate nucleus, which regulate body weight and appetite. These neurons are extremely efficient at synaptic integration, coordinating hormonal signals and excitatory synaptic inputs in order to modulate neural firing (Branco et al., 2016). As such, different types of short-time plasticity have been described during food deprivation in both AgRP and POMC neurons. On one hand, AgRP neurons have been shown to exhibit a short-term type of plasticity which involves presynaptic positive feedback of AMP-activated protein kinases (Yang et al., 2011), which in turn results in the increase of excitatory input onto AgRP neurons determining their activation during periods of fasting. Interestingly, fasting has been shown to increase the number of dendritic spines in AgRP neurons through a mechanism that require the activation of postsynaptic NMDARs (Liu et al., 2012). In fact, the growth of new synaptic contacts is consistent with an observed increase in the frequency, but not the amplitude, of AMPAR-mediated transmission. In contrast, POMC neurons in the arcuate nucleus control satiety and glucose metabolism through a fasting-dependent depression with a clear postsynaptic locus of expression (Suyama et al., 2017). As such, fasting-dependent depression of POMC neurons involves the reduction of AMPAR-mediated amplitude, but not frequency, explained by a switch in AMPAR subunit composition (Suyama et al., 2017). Intriguingly, AMPAR-mediated current rectification measurements showed that feeding increases GluA2-lacking receptors in POMC neuros through a NMDAR-independent mechanism (Liu et al., 2012).

Magnocellular hypothalamic neurons controlling blood pressure, blood volume, and Na^+^ balance also undergo experience and activity-dependent plasticity. Chronic salt-loading stimulation produces an increase in GluA1 protein expression level, subsequently potentiating AMPAR-mediated current amplitude. In addition, an increase in the frequency of AMPAR-mediated responses was also observed in parallel to the growth of glutamate release sites, which led to the formation of new synapses enriched in highly labile Ca^2+^-permeable GluA1 receptors, highly dependent on continuous dendritic protein synthesis (Di et al., 2019). In turn, osmotic activation of the hypothalamus-neurohypophysial system induces changes in glutamatergic receptors. Water deprivation increases the density of GluN1 in the SON AVP and OXT neurons (Meeker et al., 1994b) accompanied by a reduction of GluN2B expression (Decavel and Currás, 1997; Currás-Collazo and Dao, 1999). Although the functional significance of this subunit switch remains unknown, a general increase in NMDARs may underlie the low activation threshold of these neurons during dehydration, believed to serve as a signal for water re-absorption in parallel to AVP release, also controlled by NMDAR activation (Busnardo et al., 2012).

Importantly, SON and PVN magnocellular neurons undergo plastic changes during lactation and milk ejection which involve a two-fold increase in AMPAR-mediated current frequency and decay kinetics, probably due to a switch in AMPAR subunit composition (El Majdoubi et al., 1996, 1997; Pak and Currás-Collazo, 2002). Furthermore, lactating rats exhibit an augmentation in neurotransmitter release, synaptic density, and shared synapses (El Majdoubi et al., 1996, 1997; Stern et al., 2000; Pak and Currás-Collazo, 2002). However, and despite the great significance of lactation for animal survival, just a few studies address the role of glutamatergic regulation in this process, highlighting the need for expanding research on this topic.

Another prominent example of glutamatergic plasticity in the hypothalamus is stress-related synaptic plasticity (Bartanusz et al., 1995; Bains et al., 2015). A single acute stressful event can increase the ratio of AMPAR- to NMDAR-mediated transmission in parvocellular neurons in the PVN due to a long-lasting decrease of NMDARs triggered by the robust secretion of corticotropin-releasing hormone (Kuzmiski et al., 2010). This mechanism for decreasing synaptic strength contrasts with AMPAR internalizatiofn which usually orchestrates synaptic depression in the hippocampus (Malenka and Bear, 2004; Citri and Malenka, 2008). These findings further support the notion that glutamatergic synapses in the hypothalamus exhibit distinct regulatory mechanisms which may involve a more active mobilization of NMDARs from their synaptic locations.

Furthermore, recent work employing novel methods of transcranial direct stimulation mimicking LTP protocols on rats indicated that these procedures stimulated GluA1 translocation in hippocampal synapses but no changes in receptor localization were observed in the hypothalamus, although an increase in S831 phosphorylation was reported in both areas (Stafford et al., 2018). These results together with the lower GluA2/GluA1 ratio observed in hypothalamic neurons, support the notion that glutamate receptors in the hypothalamus may exhibit distinct trafficking and functional properties that are likely to influence plasticity in this brain area.

According to this, certain hypothalamic neurons have been shown to express negligible levels of GluN2A and GluN2B (Aubry et al., 1996), which suggests that glutamatergic transmission may directly influence hypothalamic neurons independently of NMDAR activation. An example is the synaptic potentiation induced by the activation of the glucagon-like peptide-1 (GLP-1) receptor in PVN neurons. Activation of the GLP-1 receptor results in an increase in excitatory synaptic strength mediated by the insertion of GluA1-containing AMPARs into the plasma membrane (Liu et al., 2017). Furthermore, somatostatin receptor activation (sst2) of mediobasal hypothalamic neurons inhibits the AMPAR component of glutamatergic synapses through a regulatory process that requires concomitant activation of NMDARs and mGluRs (Peineau et al., 2003). The need for the combined action of sst2, NMDARs and mGluRs to effectively depress AMPAR-mediated transmission highlights the heterogeneous nature of hypothalamic neurons, which in addition to glutamatergic inputs coordinate the function of various neurohormones and neuropeptides to generate an integrative response.

**FIGURE 2:**
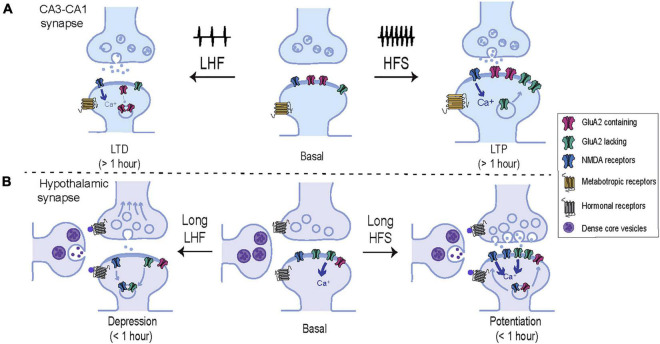
Comparative representation of the molecular mechanisms underlying plasticity in hippocampal and hypothalamic synapses. (A) Schematic representation of the model CA3-CA1 synapse. High frequency stimulation (HFS) induces calcium entry trough NMDARs activating intracellular signaling cascades that drive new AMPARs including GluA2-lacking receptors, into the synaptic membrane. These changes result in a long-lasting potentiation of the synaptic strength and an increase in spine volume. In contrast, low frequency stimulation (LFS) induces a moderate entry of intracellular calcium which drives AMPARs out from the plasma membrane, weakening synaptic strength and decreasing the spine volume. (B) Schematic representation of a hypothalamic synapse. In addition to glutamatergic inputs, hypothalamic synapses are heavily influenced by hormonal secretion from neighboring peptidergic neurons. In contrast to classical plasticity protocols in the hippocampus, hypothalamic synapses commonly exhibit short-term adaptions in response to prolong and low frequency patterns of activity. Short-term potentiation of synaptic strength can be achieved by activation of postsynaptic NMDAR and an increase of synaptic AMPARs, enriched in GluA2-lacking subunits. In addition, NMDARs can be rapidly recruited at synaptic localizations in parallel to presynaptic changes. On the other hand, hypothalamic synapses undergo short-term synaptic depression in response to low frequency stimulation by several mechanisms which may involve the activation of peptidergic or NMDA receptors, that drive the removal of synaptic GluA2-lacking AMPARs, NMDARs, and reduce glutamate release probability.

In summary, there are many evidences indicating that hypothalamic neurons undergo plastic events in which AMPAR modifications in terms of subunit composition, post-translational modifications or subcellular localization are required (summarized in Table 1). Nevertheless, the exact mechanisms involved in these processes are not as well understood as in hippocampal synapses, thus undeniably more research on this topic is needed. A conclusion drawn from the data already available is that although hypothalamic plasticity events may be shorter-lived that in the hippocampus (Abraham et al., 2002), they commonly involve AMPAR trafficking in response to activity-dependent changes usually, but not always, via NMDARs activation (see model in Figure 2).

**TABLE 1:** Summary of AMPAR and NMDAR modifications during plastic events reported in the hypothalamus.

Hypothalamic region	Cellular type	Adaptation type	Locus of expression	Synaptic modification	Bibliography
PVN	Parvocellular neurons	Depression	Presynaptic	Glutamate release reduction	Marty et al., 2011

Arcuate Nucleus	AgRP-expressing neurons	Potentiation	Presynaptic Postsynaptic	Glutamate release increase Increase NMDARs activation Increase AMPARs number	Yang et al., 2011; Liu et al., 2012; Branco et al., 2016

Arcuate Nucleus	POMC neurons	Depression	Postsynaptic	Decrease GluA2-lacking AMPARs	Suyama et al., 2017

SCN	SCN neurons	Potentiation	Postsynaptic	Increase NMDAR activation	Colwell, 2001; Pennartz et al., 2001

SON	Magnocellular neurons	Potentiation	Presynaptic Postsynaptic	Glutamate release increase Increase GluA2-lacking AMPARs	Di et al., 2019

SON	OXT-AVP Magnocellular neurons	Potentiation	Postsynaptic	Increase NMDAR number NMDAR subunit switch (↓GluN2B)	Meeker et al., 1994b; Decavel and Currás, 1997; Currás-Collazo and Dao, 1999

SON-PVN	OXT-AVP Magnocellular neurons	Potentiation	Postsynaptic Presynaptic	AMPAR subunit switch Glutamate release increase	El Majdoubi et al., 1996, 1997; Stern et al., 2000; Pak and Currás-Collazo, 2002

SON-PVN	Sst2 receptor-expressing neurons	Depression	Postsynaptic	Internalization AMPARs Cocomitant activation of NMDARs and mGluRs	Peineau et al., 2003

PVN-VMH	Androgen and strogen receptor-expressing neurons	Potentiation	Postsynaptic Presynaptic	Increase AMPARs (GluA1-3) GluA2/3 increase higher in females Glutamate release increase	Diano et al., 1997; Schwarz et al., 2008

PVN	Parvocellular neurons	Potentiation	Postsynaptic	Decrease NMDARs number	Kuzmiski et al., 2010

PVN	CRH-expressing neurons	Potentiation	Postsynaptic	Increase GluA2-lacking AMPARs	Liu et al., 2017

## AMPAR-Mediated Modulation of Homeostatic Functions

It is now clear that vital homeostatic functions regulated by hypothalamic circuits depend on the activation of AMPARs and exhibit plastic properties similar to those described in hippocampal and cortical areas. A classic example is the involvement of AMPARs in the control of gonadotropin-releasing and lutein hormones in female animals in an estradiol-dependent manner (Ping et al., 1997). Gonadotropin is released in the neurohypophysial system where stimulates the production of follicle-stimulating and luteinizing hormones, which control the proper balance of hypothalamic-pituitary-gonadal axis. In fact, gonadal steroid receptors have been found localized in glutamate receptors-expressing neurons in the hypothalamus where they affect excitatory transmission by regulating AMPAR content in a gender-specific manner (Diano et al., 1997). As such, whereas GluA1 levels equally increase in males and females, females exhibited a two-fold higher rise of GluA3/GluA2-containing AMPARs in response to estradiol (Diano et al., 1997). Moreover, estradiol induces formation of hypothalamic dendritic spines that shapes developmental sex differences by enhancing glutamate release and promoting AMPAR reorganization in hypothalamic connections (Schwarz et al., 2008).

Another important homeostatic role of the hypothalamus is the orchestration of the circadian clock, which is ultimately regulated by the SCN where photic stimulation induces phase-shifts, which in turn, control firing patterns related to pacemaker’s oscillations. Glutamate application in the SCN was described to depolarize membrane potentials (Meijer et al., 1993) mainly through NMDARs activation (Colwell, 2001), even though both NMDA and non-NMDA receptors contribute to neuronal depolarization (Michel et al., 2002). Photic stimulation triggers glutamate release from the synaptic terminals of retinal ganglion cells within the SCN, where activate postsynaptic AMPARs and NMDARs which increase calcium influx and recruit intracellular signaling cascades associated to long-term synaptic plasticity (Mikkelsen et al., 1995; Ding et al., 1997; Meijer and Schwartz, 2003). The role of NMDARs in light-induced phase-shift has been demonstrated by the light-dependent adaptation of NMDA-dependent calcium transients, which are larger and longer during the night (Colwell, 2001; Pennartz et al., 2001). In contrast to the well-defined role of NMDARs in this process, AMPAR function is unclear since their activity seems independent of circadian rhythms, although their activation leads to the increase of calcium influx in SCN neurons (Michel et al., 2002). Furthermore, exogenous AMPA application induces phase delays of locomotor activity and phase-shifts in the core clock gene *Per1* both *in vitro* an *in vivo* indicating AMPARs play a role in the entrainment of the circadian rhythms (Mizoro et al., 2010).

Furthermore, AMPAR-mediated transmission has been shown to mediate osmoticchanges associated to feeding (Hettes et al., 2003). As such, early studies in rats employing intracranial injections of AMPAR agonists and antagonists promoted or inhibited feeding depending of the targeted areas. Several studies have revealed how CNQX and NBQX are able to induce feeding in a dose dependent manner when injected in perifornical hypothalamic regions or PVN while AMPA injection induce feeding when injected into the lateral hypothalamus (Hettes et al., 2003, 2010).

Aligned with this observation, food intake and food restriction has been suggested to distinctly regulate AMPARs in different parts of the brain. For example, a short-term high fat diet has been shown to decrease GluA1 and GluA2 expression as well as GluA1 Ser845 phosphorylation levels in the hypothalamus (Liu et al., 2021). In contrast, administration of DNQX or blocking specifically GluA1 subunit in the nucleus accumbens induces feeding (Carr et al., 2009), suggesting a model where food restriction specifically promotes GluA1 expression in this region. In contrast, the manipulation of the hypothalamic neuropeptide melanin-concentrating hormone (MCHR1) in the nucleus accumbens, induces feeding behaviors in parallel to a reduction of GluA1 surface expression, mEPSC amplitude and lower GluA1 phosphorylation levels (Sears et al., 2010). This set of data indicates that AMPAR activation and inactivation in different and overlapping nuclei is sufficient to induce feeding behaviors, suggesting that the regulated switch of AMPAR subunit composition and the modulation of AMPAR number at the postsynaptic site may underlie metabolic control. More recently, the implementation of Cre-recombinase-enabled and cell-specific mapping techniques in mice have allowed elegant studies to reveal an unknown excitatory drive from the PVN to AgRP-expressing neurons in the arcuate nucleus (Krashes et al., 2014). Interestingly, leptin-mediated signaling has been shown to modulate NMDARs and AMPARs to influence neuronal excitability and synaptic plasticity in the hippocampus (reviewed in Gavello et al., 2016), although its role in modulating glutamatergic transmission in the hypothalamus remains elusive.

## Hypothalamic AMPAR- Mediated Transmission in Pathology

As aforementioned, glutamatergic transmission influences practically all autonomic and homeostatic responses orchestrated in the hypothalamus including stress, energy and electrolyte balance, circadian rhythms, blood pressure, lactation, and fertility. Given the various roles of AMPAR-mediated transmission in the hypothalamus, it is tempting to hypothesize that alterations of its function may trigger pathological conditions related to brain and body homeostasis maintenance. As such, AMPARs has been shown to facilitate stress-evoked autonomic responses (e.g., arterial blood pressure and heart rate) (Busnardo et al., 2013), and an enrichment of GluA2-lacking AMPARs contributes to the increased excitability of PVN presympathetic neurons related to hypertension (Li et al., 2012). Furthermore, the involvement of AMPAR in the maintenance of normal neurological function suggests that dysregulations of their trafficking, phosphorylation or subunit composition may be associated with cognitive and behavioral impairments as varied as anxiety, depression, ischemia, intellectual disability, neurodegenerative conditions, drug addiction or social deficits (Krugers et al., 2010; Kuniishi et al., 2020; Zhang and Bramham, 2020; Babaei, 2021; Ge and Wang, 2021; Wu et al., 2021). In addition, several research works have demonstrated that positive modulators of AMPARs leads to antidepressant effects improving behavioral, neurochemical and glutamate-transmission deficits in perinatal stressed rats (Andreasen et al., 2013, 2015; Morley-Fletcher et al., 2018). In contrast, reduction of AMPAR transmission may underlie anxiety and stress (Alt et al., 2006; Andreasen et al., 2015; Li et al., 2017; Hasegawa et al., 2019). Increased stress vulnerability has been also related to GluA2 trafficking alterations in a GluA2 mutant (GluA2 K882A) with disrupted PKC-dependent phosphorylation and exacerbated anxiety (Ellis et al., 2017), highlighting the relevance of AMPAR-mediated transmission in stress regulation and emotional responses.

Interestingly, recent cumulative evidence points to the dysregulation of AMPAR trafficking as a major culprit of cognitive and social disorders, such as autism spectrum disorder (ASD) (Danesi et al., 2019). As such, *Frmp1* KO mice, characterized by neurological and behavioral ASD-like symptoms and repetitive behaviors, show reduced levels of PKC_ε_, AMPAR phosphorylation deficits and aberrant recycling of GluA2-containing receptors through a process that impacts the development of OXT neurons in the PVN (Marsillo et al., 2021). Stimulation of PKC_ε_ at early stages of postnatal development reduced the hyper-anxiety and social behavior impairments, and increased GluA2 recycling (Marsillo et al., 2021). Another example of the importance of maintaining adequate phosphorylation levels of AMPARs is the data from the *Grip1/2D* KO mice which present increased social interactions and augmented levels of GluA2 phosphorylation (Han et al., 2017). These results together with previous observations from a Grip1 gain-of-function mutant (Mejias et al., 2011) indicate that preserving the ratio of GluA2-containing receptors play an important role in the modulation of social behaviors.

Moreover, a developmental misbalance of excitation/inhibition of neural circuits has been identified as a common underlying mechanism of ASD (Polleux and Lauder, 2004; Orekhova et al., 2007). In fact, a general reduction of AMPAR density has been found in postmortem brain samples of ASD patients particularly in the cerebellum and the prefrontal cortex (Purcell et al., 2001) with no reported data for the hypothalamic region. Importantly, a recent study also examined glutamatergic transmission in the cortex of two different ASD models: a contactin-associated protein-like 2 gene (*Cntnap2*) KO (Gdalyahu et al., 2015) and a prenatal exposure of valproic acid-induced mouse model (VPA mice). Both animal models exhibit alterations in their glutamate receptors expression patterns in the cortex: however, while *Cntnap2* KO mice displayed reduced glutamatergic expression and activity, VPA-exposed mice showed an increase in glutamatergic receptors, and nonetheless they both exhibited similar autism-like behaviors (Kim et al., 2019). Intraperitoneal injection of AMPAR agonist in the *Cntnap2* KO or AMPAR antagonist in the case of the VPA-exposed model, restored social behavior suggesting an important role for AMPARs in the physiopathology of the disease. Interestingly AMPAR agonists/antagonists had no effect treating repetitive behaviors, which have been associated to NMDARs abnormal function (Lewis and Kim, 2009; Archer and Garcia, 2016). According to this, social deficits and repetitive behavior were restored in a VPA-exposed rat model, characterized by ASD-like symptoms and impaired NMDAR-dependent LTD, after the administration in the lateral amygdala of D-cycloserine (DCS), a cognitive enhancer that increases NMDARs function (Wu et al., 2018). Nonetheless, DCS action also impacted AMPARs by facilitating the removal of GluA2-containing AMPARs, and enabling NMDAR-dependent LTD in the lateral amygdala. These findings expose the importance of NMDAR and AMPAR balance in the development and clinical manifestation of neurological disorders related to social behaviors. Furthermore, the current lack of information regarding the status of hypothalamic glutamatergic function in the context of pathological conditions highlights the need of intensifying research efforts on this topic.

## Conclusion

The significance of glutamatergic, and particularly AMPAR-mediated transmission, in hypothalamic function is just starting to emerge. Selective targeting of AMPARs in specific neurons within distinct hypothalamic nuclei could be the foundation of novel therapies for disorders as varied as hypertension (Li et al., 2012), feeding disorders (Florent et al., 2020), circadian clock dysregulation (Rijo-Ferreira and Takahashi, 2019) and social disorders (Carlson, 2012; Kim et al., 2019). In order to accomplish this, more research will be needed to understand the role and regulatory mechanisms of glutamatergic receptors in hypothalamic synapses. Of particular interest will be to elucidate the role of AMPA and NMDA receptors in basal synaptic transmission, and the dynamic processes involved in the various types of hypothalamic plasticity. Also basic knowledge as the identification of the drivers of the key signaling pathways involved in long-term synaptic changes as well as the scaffold and auxiliary proteins implicated in distinct hypothalamic synapses will be fundamental to unveil the role of glutamatergic function in brain and body homeostasis.

## Author Contributions

MR and BA wrote the article. MM made the figures. SJ edited the article. All authors contributed to the article and approved the submitted version.

## Conflict of Interest

The authors declare that the research was conducted in the absence of any commercial or financial relationships that could be construed as a potential conflict of interest.

## Publisher’s Note

All claims expressed in this article are solely those of the authors and do not necessarily represent those of their affiliated organizations, or those of the publisher, the editors and the reviewers. Any product that may be evaluated in this article, or claim that may be made by its manufacturer, is not guaranteed or endorsed by the publisher.
